# Ocular Tonometry and Sporadic Creutzfeldt - Jakob Disease (sCJD): A Confirmatory Case-Control Study

**DOI:** 10.9734/BJMMR/2014/7247

**Published:** 2014-04-30

**Authors:** Zoreh Davanipour, Eugene Sobel, Argyrios Ziogas, Carey Smoak, Thomas Bohr, Keith Doram, Boleslaw Liwnicz

**Affiliations:** 1 Department of Neurology, Loma Linda University School of Medicine, Loma Linda, CA 92350, USA; 2 Department of Neurology, Keck School of Medicine, University of Southern California, Los Angeles, CA 90033, USA; 3 Department of Preventive Medicine, Keck School of Medicine, University of Southern California, Los Angeles, CA 90033, USA; 4 Department of Internal Medicine, Loma Linda University School of Medicine, Loma Linda, CA 92350, USA; 5 Department of Pathology, Loma Linda University School of Medicine, Loma Linda, CA 92350, USA

**Keywords:** Sporadic Creutzfeldt-Jakob disease (sCJD), prion diseases, intraocular pressure (IOP) test, risk factors, iatrogenic transmission, eyes, ophthalmology, case-control study, neuroepidemiology

## Abstract

**Aims:**

To evaluate the hypothesis that sporadic Creutzfeldt-Jakob disease (sCJD) may be transmitted through ocular tonometry.

**Background:**

The infectious agent of sCJD may be present in the cornea prior to clinical symptoms. Cornea infectiousness has been documented by cornea transplants in guinea pigs and humans. sCJD is resistant to complete inactivity by conventional sterilization techniques. Thus contact tonometry equipment is not disinfected sufficiently to kill sCJD. We previously hypothesized that contact tonometry is a sCJD risk factor.

**Study Design:**

Population-based case-control study.

**Place and Duration of Study:**

Department of Neurology, School of Medicine, Loma Linda University, Loma Linda, CA, USA; 4 years.

**Methodology:**

An 11-state case-control study of pathologically confirmed definite sCJD cases, individually matched controls, and a sample of control surrogates was conducted. Ocular tonometry histories were obtained from case-surrogates, controls, and a sample of control-surrogates.

**Results:**

The odds ratio (OR) for ever vs never having had an ocular tonometry test was statistically significant for matched and unmatched analyses for 15 through 3 years prior to disease onset, using both control self-responses and control surrogates: ORs were ∞ and 19.4 with 1-sided P-values <0.0001 and 0.003 and ORs=∞ and 11.1 with 1-sided P-values <0.003 and 0.02, respectively. ORs increased as the number of tonometry tests increased during this age period: trend test, 2-sided P-value < 0.0001. For ≥5 vs <5 tonometry tests, the OR was 5.8 (unmatched) and 3.7 (matched), 2-sided P-value<0.00005. Respondents generally could not specify the type of tonometry. There was no indication of increased tonometry testing among cases within 2 years of disease onset.

**Conclusions:**

The *a priori* hypothesis was supported. Contact tonometry, preferred by ophthalmologists, may be capable of transmitting sCJD. Consideration should be given to using disposable instrument covers after each use. The use the disposable covers or non-contact tonometry is preferable in the absence of effective disinfectant processes at this time.

## 1. INTRODUCTION

In 1984, the New England Journal of Medicine published a letter presenting the glaucoma testing results of a study of sporadic Creutzfeldt-Jakob disease (sCJD) and various exposures [1]. This study found that glaucoma testing may be a risk factor for sCJD. Prior to 1984 Duffy et al. reported the occurrence of sCJD in an individual who had received a corneal transplant from a subject with definite sCJD [2]. In 1977, Manuelidis et al. reported the transmission of sCJD from the cornea of infected guinea-pigs to healthy guinea-pigs via the anterior chamber of the eye [3]. Subsequently, there have been a few more reports of cornea transplant patients developing sCJD when the donor had either definite, probable or possible sCJD [4,5]. Glaucoma tests are usually performed using intraocular pressure (IOP) Goldmann tonometry, during which the equipment actually touches the cornea. Furthermore, the equipment cannot be disinfected after each use to completely “kill” the sCJD infectious agent (prion, PrP^Sc^) [6,7]. This is due to resistance of the agent to complete inactivation by conventional sterilization techniques. Thus, we hypothesized that intraocular pressure tests, intraocular tonometry, would be a risk factor for sCJD. In our initial study, we did find that a history of glaucoma testing was significantly associated with sCJD [8].

## 2. MATERIALS AND METHODS

This study was approved by the Institutional Review Board (IRB) of the Loma Linda University School of Medicine. The study participant subjects have signed the IRB approved informed consent form.

### 2.1 Study Subjects

The present report is based on an 11-state case-control study of neuropathologically confirmed sCJD. The 11 states in the study were chosen because of their size; combined, they contain about 40% of the US population. The process of identifying subjects with a neuropathological confirmation of CJD has been detailed elsewhere [9]. Neuropathologically confirmed CJD cases were identified through systematic inquiries of hospitals and neuropathologists, and through the use of death certificates. All cases had been diagnosed between 1979 and 1990. The study neuropathologist (BL) reviewed the neuropathology reports and/or slides and tissue blocks for 189 of these cases. One hundred sixty-two (162) were confirmed to have had definite CJD. Thirty-two families declined to participate and another 10 families could not be located. Thus, families for 120 cases participated and provided surrogate information. The 10 familial CJD cases, defined as a case who had a blood relative with at least suspected CJD, were excluded from this analysis because familial CJD is overwhelmingly of genetic, not environmental, origin. If such cases had been included in the analyses, the odds ratio (OR) estimators would have therefore been somewhat biased towards one. Thus, only sporadic CJD cases were used in the analyses.

Controls were obtained by random-digit-dialing. Matching criteria were date of birth (10 years earlier than to 2 years after the case’s birth), gender, ethnicity (African-American, White, Hispanic, Asian) and geographic area of residence 2 years prior to the onset of disease. The study protocol called for up to 2 controls per case. One control was found for 56 cases and 2 controls for 29 cases. There were no controls for 25 (23%) cases because of study funding limitations associated with the time needed to recruit matched controls by random digit dialing.

It was our objective to determine whether our earlier findings [8] were replicable. These two studies are completely independent. No case or control from the earlier study was in the present study. In fact, the states from which cases were obtained in each study were non-overlapping.

### 2.2 Data Collection Interviews

Due to the nature of the disease (i.e., progressive dementia and death usually within one year), the most knowledgeable surrogate was interviewed for each case. Controls were directly interviewed. However for a subsample of controls, a knowledgeable surrogate was also interviewed. All interviews were conducted by telephone, which provided an efficient and feasible data collection method for a study which covered a large geographical area. Interviewees were sent materials describing the categories of information of interest so that they could prepare for the interview. The interviewers were blinded as to the study hypotheses, disease of interest and case-control status. They knew only that the study was health-related and that sometimes the interview concerned the person being interviewed and sometimes it concerned another person. The questionnaire was quite detailed and covered many areas, including diet, medical problems, occupation, contact with animals, travel, glaucoma testing. Interviewees were unaware of the study hypotheses. Controls and control-surrogates were unaware of the disease being studied.

### 2.3 Ocular Tonometry Exposures

The questionnaire was similar to the one used in our original sCJD study [8,10]. It was, however, more focused. Exposure data were obtained for three periods for cases: birth through 14 years of age; age 15 through 3 years prior to symptom onset; within 2 years prior to symptom onset. For the individually matched controls, corresponding periods were used. For each period, interviewees were asked about the occurrence of one or more glaucoma tests and the number of ocular tonometry. Information concerning having contact and non-contact ocular tonometry was requested. However, over 50% of the respondents (case-surrogates, controls and control-surrogates) could not specify contact versus non-contact ocular tonometry.

### 2.4 Statistical Analyses

Because this is a confirmatory study, we have chosen to use one-sided statistical tests (P-values) for the odds ratio estimates. The P-value for a 2-sided test is simply twice the P-value for the corresponding 1-sided test. Exact conditional logistic regression was used for estimating the P-values and 95% (2-sided) confidence intervals for the standard odds ratio estimates for the *ever* versus *never* (dichotomous) comparisons [11]. Odds ratio estimation for the exposure index based on the number of times a subject had had a glaucoma test was performed using conditional logistic regression with 2-sided P-values [12]. Thus, the case-control matching was retained in all these analyses. We also performed unmatched (unconditional) logistic regression analyses for comparison purposes and for a 1-degree of freedom trend test [12].

As mentioned above, respondents generally were uncertain as to the type of tonometry equipment (pressure vs puff) used. We therefore have conducted all analyses without attempting to differentiate between the two types of equipment.

## 3. RESULTS

### 3.1 Study Subjects

Forty-eight (56%) of the cases with a control were men. There was very little difference in the mean or median ages at onset between men and women (63.0 vs 62.1; 62.1 vs 63.2). The standard deviations were the same: 8.4 years. The cases without a matched control were, on average, 4 years older at onset and had a smaller onset age standard deviation than the cases with at least one control. The cases without a matched control also had a slightly shorter mean duration of disease with a smaller standard deviation: mean durations of 7.3 vs 8.7 months; standard deviations of 5.6 vs 11.5 months. The differences in the standard deviations were due to a few cases, among those with controls, who had a long duration of illness.

### 3.2 Ocular Tonometry Test Histories

Information about ever having had an intraocular pressure (IOP) test, ocular tonometry from birth through age 14 was missing for 41% of the cases and 47% of the controls using surrogate data, but only for 3.5% of the controls using self-reported data (Table 1). For the age period 15 through 3 years prior to disease onset, missing information was minimal (8%, 12%, and 3%). For the period within 2 years prior to onset, the missing information percentages were 9%, 1%, and 12%). The rates of ever having had a glaucoma test during the initial period (birth through age 14) were quite low, but were substantial during the other two periods (Table 1). Information on the number of glaucoma tests within a specific period was missing somewhat more often for cases than for control self-responses: 16%/15% vs 9%/10%. For control-surrogate data, the rates of missing information about the number of glaucoma tests were over 50%. The absolute and relative frequencies of the categorized data are provided in Table 1. The rates of missing data are essentially identical for cases with and cases without controls (data not shown).

### 3.3 Dichotomous (Ever vs Never) Exposure Odds Ratios

Table 2 provides the odds ratio estimates for glaucoma tests using the dichotomy *ever* vs *never* by age-period. The data for the period birth through age 14 were too sparse for the use of matched analyses or control surrogate data. The analyses using control self-responses clearly indicate a significantly increased risk of CJD among those who had ever had a glaucoma test from age 15 through 3 years prior to disease onset. The data for the age period 15 through 3 years prior to disease onset have no discordant pairs or triples with the case non-exposed. There were 10 discordant pairs and 6 discordant triples (one control exposed and one not exposed) with the case exposed. This leads to an OR estimate of ∞, P<0.0001. The analysis using the control surrogate data are based on only 32 cases and 34 controls. The results are in agreement with the self-reported control data, but the estimates are unstable because of the relatively small sample size.

For the period within 2 years of onset, there is no indication of an increased risk associated with having had a glaucoma test in that period. For this period, the OR estimates are 1.0 or lower, and are not statistically significant. The relative frequencies of ever having had a glaucoma test during the middle period and last period were about equal among cases, but the proportion of controls ever having had a test increased from 84% to 96%, perhaps because decreases in vision often come with older age.

### 3.4 Odds Ratios Based on Number of Glaucoma Tests

Table 3 presents the results of an analysis using specific categories of the number of glaucoma tests in the middle and last periods. The cut-points are different within each period because of the number of subjects with multiple tests, in part certainly due to the greater duration of the middle period (age 15 through 3 year prior to onset) compared to the last period (within 2 years of onset). For the middle period there is a significantly increased risk associated with each of the categories 1–4, 5–10 and > 10. There is also a clear trend in the increase of the odds ratio estimate with increased number of glaucoma tests (P < 0.0001).

Finally, Table 4 presents the results for the period age 15 through 3 years prior to onset for the number of glaucoma tests with a single cut-point between 4 and 5 tests. Both the matched and unmatched analyses (≥5 vs < 5 tests) are provided using control self-responses. The odds ratio estimates are 5.8 and 3.7 and are highly statistically significant.

## 4. DISCUSSION

### 4.1 *A Priori* Nature of the Study Hypothesis

The hypothesis that ocular pressure tonometry is associated with the occurrence of CJD was an *a priori* hypothesis based on our previous case-control study of sCJD and on ours and others hypothesis on the transmission of sCJD as described in the Introduction. Cornea is one of the eye structures which may contain PrP^Sc^ prior to the clinical onset of disease. There have been demonstrated cases of recipients receiving infected cornea transplants and within a few years developing sCJD [2–5].

Lim et al. [6] have demonstrated that a person who has a contact glaucoma test (the procedure usually preferred by ophthalmologists and optometrists) will shed some cells onto the equipment. The equipment is not disinfected sufficiently between uses to “kill” the CJD infectious agent [6,7]. The infectious agent is resistant to complete inactivation by conventional sterilization techniques. Head et al. [13] have investigated the distribution of prions in the eyes of one patient with sCJD and two patients with vCJD. sCJD and vCJD were confirmed pathologically. PrP^Sc^ was not detected in the cornea of the examined eyes. The authors state, however, that because (1) transmission through corneal transplants has been documented and (2) the lack of sufficient sensitivity of the assay they used to detect PrP^Sc^, the lack of detection “cannot be taken as evidence for the absence of infectivity” of the cornea.

### 4.2 Study Findings

In this confirmatory case-control study of sCJD, we have found that ocular tonometry, particularly between age 15 through 3 years prior to disease onset, may be a risk factor for sCJD. We have also shown a dose-response effect in that the larger the number of glaucoma tests the higher the relative risk of disease. Unfortunately, the respondents most often could not differentiate between the puff (non-contact) and the contact tonometry when queried about the history of each type of test. Thus, we simply analyzed tonometry tests without differentiating between types of equipment used. Further studies in countries with health care systems which have national computerized records of glaucoma tests and for which the types of equipment can be determined would be fruitful.

The estimated ORs for the period within 2 years of disease onset were 1.0 or below. Thus, errors in estimating the age or time of onset of disease are unlikely to have resulted in an upward bias in the results for the period age 15 through 3 years prior to disease onset.

### 4.3 Multiple Comparisons

There are no multiple comparison problems complicating the interpretation of our results. The *a priori* hypothesis was based on the results of a previous study combined with knowledge of the infectious nature of the cornea from a donor with sCJD. Furthermore the study protocol stated that this particular hypothesis would be tested.

### 4.4 Control Surrogates

Analyses using control-surrogates generally support the results of the analyses using the control self-responses (Table 2), even though the control-surrogate sample size is small.

### 4.5 European Study Design and Finding

Zerr et al. [14], as part of the European CJD surveillance project, analyzed “ophthalmological tests” and reported no increased risk of CJD associated with ever having had an ophthalmological test. Zerr et al. did not differentiate between types of ophthalmological tests, nor did they list the tests included in the analyses. No information concerning ocular tonometry *per se* was provided. We note that 72% of their 405 controls had a neurologic disease and 25% were hospital controls with non-neurological diseases. There were only 8 population controls. The percentages of both cases and controls in the Zerr et al. study who had ever had an ophthalmological test were 50% and 55%, respectively. This is significantly lower than in our study, where percentages for the age period 15 through 3 years prior to onset were 99% and 84%. Perhaps ocular tonometry was not considered an ophthalmologic test. In addition, ophthalmologic problems are not uncommon among patients with stroke and other neurologic diseases [15–21]. It would therefore appear that controls with neurologic disease are inappropriate for investigating a possible risk of sCJD associated with tonometry or ophthalmologic tests in general.

### 4.6 Bias Protection

The rationale for and critique of case-control epidemiologic studies, and areas for improvement in such studies of CJD have been detailed elsewhere [22]. de Pedro Cuesta et al. [23] have also described some potential well-known biases in epidemiologic research. In particular, there are three potential biases of relevance here. We discuss these potential biases and how they were dealt with in the present study below.

#### 4.6.1 Use of Hospital controls rather than non-hospital controls living in the same general area as the case

No control was hospitalized. All controls lived in the same geographic area (same area code and prefix) as the corresponding case.

#### 4.6.2 Use of control exposure information related to time periods of different lengths from the corresponding case time periods

The exposure time period lengths for cases and controls were the same. The ages of the cases and controls were the same for each of the three (3) time periods utilized for exposure data.

#### 4.6.3 Obtaining exposure information from case-surrogates and directly from matched controls

Exposure information was obtained from case-surrogates and directly from matched controls. However, with great effort, it was possible to recruit control-surrogates for 34 of the 114 (30%) controls. The control-surrogate data were analysed in Tables 1 and 2. In order to assist case-surrogates to provide more complete and accurate information, they were encouraged to discuss specific exposure inquiries with other people who also knew the case well. Note that all cases were deceased. For controls, control-surrogates were requested not to discuss exposure questions with the control.

## 5. CONCLUSION

The study was designed and conducted to minimize problems often associated with case-control studies, particularly when the cases are mentally incapacitated or deceased. The findings indicate that ocular tonometry may be an important iatrogenic method of transmission of the infectious agent for sCJD.

We note that disposable protective covers and disposable tonometer tips, which essentially eliminate any risk associated with contact tonometry, are available but are not yet commonly used [24–26]. The British Royal College of Ophthalmologists in a document dated May 2004, evidently no longer available on line, recommended that contact tonometry equipment be wiped and disinfected after each use. They further recommended that disposal heads or shields or Tono-Pen Tip Covers be used, but only when a subject either has or may have or be possibly genetically susceptible to CJD. See [27] for an update. The update suggests use of disposable covers when the subject is “known to have, or under suspicion of having, CJD”, including “neurological diseases such as dementia of unknown etiology”. These recommendations may not be sufficiently strict.

## Figures and Tables

**Table 1:** Distribution of tonometry test data by period: Number and Percent

VARIABLE	PERIOD	CATEGORY	CASES	CONTROLS	
				SELF-REPORT	SURROGATE
			N=110	N=114	N=34*
Ever vs Never	Birth to Age 14	No	59 (91%)	108 (98%)	18 (100%)
		Yes	6 (9%)	2 (2%)	0 (0%)
		Missing	45 (41%)**	4 (4%)**	16 (47%)**

	Age 15 to 3 Years Prior to Onset	No	1 (1%)	18 (16%)	3 (10%)
		Yes	100 (99%)	93 (84%)	27 (90%)
		Missing	9 (8%)	3 (3%)**	4 (12%)**

	Within 2 Years Prior to Onset	No	5 (5%)	5 (4%)	1 (3%)
		Yes	95 (95%)	108 (96%)	29 (97%)
		Missing	10 (9%)**	1 (1%)**	4 (2%)**

Number of Tests	Age 15 to 3 Years Prior to Onset	Zero	1 (1%)	18 (17%)	3 (20%)
		1–4	17 (19%)	31 (30%)	0 (0%)
		5–10	38 (41%)	33 (32%)	3 (20%)
		> 10	36 (39%)	22 (21%)	9 (60%)
		Missing	18 (16%)**	10 (9%)**	19 (56%)**

	Within 2 Years Prior to Onset	Zero	5 (5%)	5 (5%)	1 (6%)
		1	20 (20%)	14 (14%)	1 (6%)
		2	57 (57%)	19 (18%)	0 (0%)
		>2	11 (11%)	65 (63%)	14 (88%)
		Missing	17 (15%)**	11 (10%)**	18 (53%)**

* Two cases had two controls each with a surrogate control. However, for neither case did both surrogate controls supply tonometry data.

** Percent is based on the total number of subjects: 110 cases; 114 controls; 34 control surrogates.

**Table 2:** Odds ratio estimates for tonometry tests using the dichotomy *ever* vs *never* by period

PERIOD	ESTIMATION PROCEDURE	ODDS RATIO ESTIMATE	95% (2-sided) CONFIDENCE INTERVAL	P-VALUE (1 sided)

Birth – Age 14	Unmatched: Control Self-Responses	5.5	1.1 – 28.1	0.02

Age 15 – 3 Years Prior to Onset	Matched: Control Self Response	∞	3.1 – ∞	< 0.0001
	Matched: Control Surrogates*	∞	0.7 – ∞	0.13
	Unmatched: Control Self-Response	19.4	2.5 – 147.9	< 0.003
	Unmatched: Control Surrogates*	11.1	1.1 – 111.1	< 0.02

Within 2 Years Prior to Onset	Matched: Control Self Response	1.0	0.2 – 5.1	0.31
	Matched: Control Surrogates*	0.3	0.01 – 4.2	0.16
	Unmatched: Control Self-Response	0.9	0.2 – 3.1	0.42
	Unmatched: Control Surrogates*	0.7	0.07 – 5.8	0.80

* Uses only the 32 cases for whom one matched control has surrogate data. No case had 2 matched controls each with surrogate data.

**Table 3:** Odds ratio estimates for tonometry tests using ordinalized data for number of glaucoma tests by period

PERIOD	ESTIMATION PROCEDURE/CATEGORIES	ODDS RATIO ESTIMATE	95% (2-sided) CONFIDENCE INTERVAL	P-VALUE (1-sided)	P-VALUE for TREND

Age 15 – 3 Years Prior to Onset	Unmatched: Control Self-Responses				
	Never	1.0			
	1–4	9.9	1.2 – 80.5	< 0.04	
	5–10	20.7	2.6 – 163.8	0.004	< 0.0001
	> 10	29.5	3.7 – 236.3	< 0.002	

Within 2 Years Prior to Onset	Unmatched: Control Self-Responses				
	Never	1.0			
	1	1.4	0.3 – 5.9	0.62	
	2	3.0	0.8 – 11.5	0.11	NS*
	> 2	0.2	0.04 – 0.7	< 0.02	

* NS = Not Significant

**Table 4:** History of 5+ tonometry tests - age 15 through 3 years prior to onset

EXPOSED STATUS	N of CASES	N of CONTROLS	NUMBER OF CONTROLS PER CASE				
			1		2		

			NUMBER OF CONTROLS EXPOSED				
			0	1	0	1	2

NO	18	49	8	2	1	1	2
YES	74	55	23	13	1	8	10
MISSING	18	10					

TYPE OF ANALYSIS	ODDS RATIO	95% (2-sided) CONFIDENCE INTERVAL	P-VALUE (1-sided)

MATCHED: CONTROL SELF-RESPONSES	5.8	2.2 – 19.1	< 0.00005
UNMATCHED: CONTROL SELF-RESPONSES	3.7	1.9 – 7.0	< 0.00005

## ABBREVIATIONS

CJD: Creutzfeldt-Jakob disease
IOP: intraocular pressure
PrP^SC^: infectious misfolded iso form Prion Protein
sCJD: sporadic Creutzfeldt-Jakob disease
vCJD: variant Creutzfeldt-Jakob disease
